# Effects of Hormone Therapy and Flavonoids Capable on Reversal of Menopausal Immune Senescence

**DOI:** 10.3390/nu13072363

**Published:** 2021-07-10

**Authors:** Nikolaos Vrachnis, Dimitrios Zygouris, Dionysios Vrachnis, Nikolaos Antonakopoulos, Alexandros Fotiou, Periklis Panagopoulos, Aggeliki Kolialexi, Kalliopi Pappa, George Mastorakos, Zoi Iliodromiti

**Affiliations:** 1 Third Department of Obstetrics and Gynecology, School of Medicine, National and Kapodistrian University of Athens, Attikon Hospital, 12462 Athens, Greece; antonakopoulos2002@yahoo.gr (N.A.); paninosrafaela@yahoo.gr (P.P.); 2 Research Centre in Obstetrics and Gynecology, Hellenic Society of Obstetric and Gynecologic Emergency, 11528 Athens, Greece; dzygour@med.uoa.gr; 3 Vascular Biology, Molecular and Clinical Sciences Research Institute, St George’s University of London, London SW17 0RE, UK; 4 Department of Clinical Therapeutics, School of Medicine, National and Kapodistrian University of Athens, Alexandra Hospital, 11528 Athens, Greece; dionisisvrachnis@gmail.com; 5 Department of Gynecologic Oncology, Metaxa Memorial Cancer Hospital, 18537 Piraeus, Greece; alexandrosfotiou92@gmail.com; 6 Department of Medical Genetics, School of Medicine, National and Kapodistrian University of Athens, 11527 Athens, Greece; akolial@med.uoa.gr; 7 First Department of Obstetrics and Gynecology, School of Medicine, National and Kapodistrian University of Athens, Alexandra Hospital, 11528 Athens, Greece; kpappa@med.uoa.gr; 8 Unit of Endocrinology, Diabetes Mellitus and Metabolism, School of Medicine, National and Kapodistrian University of Athens, Aretaieion Hospital, 11528 Athens, Greece; mastorakg@gmail.com; 9 Neonatal Department, School of Medicine, National and Kapodistrian University of Athens, Aretaieio Hospital, 11528 Athens, Greece; ziliodromiti@yahoo.gr

**Keywords:** flavonoids, isoflavones, hormone replacement therapy, menopause, immune senescence, inflammation, inflammatory cytokines, immune system

## Abstract

Menopause, probably the most important natural change in a woman’s life and a major component of female senescence, is characterized, inter alia, by cessation of ovarian estrogen and progesterone production, resulting in a gradual deterioration of the female immune system. Hormone replacement therapy (HRT) is used in postmenopausal women to relieve some of the peri- and postmenopausal symptoms, while there is also evidence that the therapy may additionally partially reverse menopausal immune senescence. Flavonoids, and especially isoflavones, are widely used for the treatment of menopausal symptoms, although it is not at present clear whether they can reverse or alleviate other menopausal changes. HRT reverses the menopausal CD4/CD8 ratio and also limits the general peri- and postmenopausal inflammatory state. Moreover, the increased levels of interleukins (IL)-1β, IL-6, and IL-8, as well as of tumor necrosis factor-α (TNF-α) are decreased after the initiation of HRT. However, some reports show no effect of HRT on IL-4, IL-10, and IL-12. It is thus evident that the molecular pathways connecting HRT and female immune senescence need to be clarified. Interestingly, recent studies have suggested that the anti-inflammatory properties of isoflavones possibly interact with inflammatory cytokines when applied in menopause treatments, thereby potentially reversing immune senescence. This narrative review presents the latest data on the effect of menopausal therapies, including administration of flavonoid-rich products, on age-associated immune senescence reversal with the aim of revealing possible directions for future research and treatment development.

## 1. Introduction

Menopause, which is characterized by the termination of the ovarian production of estrogen and progesterone and is considered to be the most important natural change in a woman’s life, is accompanied by a large number of correlated changes. In order to alleviate some of these, such as menopausal vasomotor symptoms and genital atrophy, and to prevent or reduce bone loss, hormone replacement therapy (HRT) is frequently used by peri- and postmenopausal women. However, there are a number of additional applications of hormone treatment (HT), in which hormones are administered for other medical purposes in humans or tested in animal models.

Flavonoids are a class of polyphenolic compounds: isoflavones are the most widespread, mainly found in legumes, grains, nuts, and vegetables, with soybeans being the richest source. They have been used successfully for the treatment of menopausal symptoms and have also shown a favorable effect on prevention and treatment of osteoporosis in postmenopausal women [1,2,3].

Another result of the menopausal transition is the senescence, or aging, of the female immune system. In this setting, a reduced immune response develops, as well as an increased inflammatory state, which condition results in the triggering or exacerbation of an array of disorders [4].

The last decade has seen the publication of several studies reporting the considerable beneficial effect exerted by female sex hormones both on postmenopausal conditions and on the immune system, especially with regard to autoimmune diseases and infections [5]. The authors, meanwhile, also demonstrated the manner in which low estrogen levels compromise the immune response, thus predisposing women to disease and infection [6,7,8,9]. A number of studies have, moreover, pointed to the contribution of ovarian sex steroid loss towards immune senescence among women, while also showing that menopausal hormone therapy (MHT) is capable of delaying several of these changes [10]. In addition, recent in vitro studies have revealed the anti-inflammatory effects of isoflavones, mentioned above, as evidenced by a reduction in the levels of inflammatory cytokines [11,12,13].

Since it is well-known that ovarian steroids modulate the immune response, it could be suggested that HRT may possibly reverse immune senescence. Nevertheless, the exact molecular pathways connecting HRT and immune senescence have not to date been elucidated. Chronic inflammation, promoted by certain lifestyle factors, is known to play a key role in human health by inducing diseases that lead to morbidity and mortality. It is of interest to note that the increased vascular inflammation [14] observed in women in early menopause, as compared to age-matched premenopausal women, is mirrored by a similar phenomenon that manifests from birth in premature newborns of both sexes [15,16], a condition which lasts throughout life [17,18,19,20].

Given that reduction of inflammation shows clear improvement in certain cases, deeper insight into the molecular pathways of the inflammatory cascade may well offer highly promising options for future treatments [21].

In this narrative review, we present the most recent data concerning the impact of hormonal menopausal therapies and isoflavones on the immune system and potential, resultant senescence reversal with the aim of revealing possible directions for future research and treatment development, while we also discuss flavonoid-rich products that may be used for pharmaceutical purposes or as functional foods.

## 2. Basic Functions of the Immune System Related to Immune Senescence

The prime function of the human immune system is to provide protection from external pathogens and to fight infection: this is accomplished via its recognition of self and nonself antigens, while it also assesses microbial threats and deals with their elimination [22]. It is, moreover, essential for minimizing any damage done to tissues, an action that inhibits the development of such disorders as autoimmune diseases. This extraordinary process of fine-tuning is made possible via the subtle interplay between the two vital strands of the immune system, namely, innate and adaptive immunity [23].

The actions of the innate immune system, which constitutes the body’s first line of defense against diseases nonspecific for any pathogen, are mediated by natural killer (NK) cells, neutrophils, macrophages, and dentritic cells. All the latter identify microbial nonself pathogens by means of pattern recognition repeaters (PRRS), of which toll-like receptors (TLR) compose the best characterized family. NK cells mediate recognition of missing or altered self through expression of an array of activating and inhibitory receptors. Macrophages and neutrophils, meanwhile, participate in the removal of pathogens via phagocytosis, whereas activation of these macrophages and neutrophils results in an inflammatory response on account of the formation of interleukin (IL)-6, IL-8, and interferon-α (IFN-α). Finally, IL-6, acting as a proinflammatory cytokine, activates IL-1 and IL-10 while also inhibiting tumor necrosis factor-α (TNF-α): it thereby stimulates the autoimmune and inflammatory process in a range of diseases. IL-8, a neutrophil chemotactic factor, is increased by oxidant stress, inducing localized inflammation by cleaving metalloproteinase molecules [24]. IFN-α, an inflammatory cytokine that may be inhibited by IL-10, induces immune dysfunction in autoimmune diseases while also mediating tissue inflammation [25].

The two types of lymphocytes of the adaptive immune response are T and B cells, which are able to recognize and respond specifically to each pathogen. B cells, or B lymphocytes, produce large quantities of antibodies specific to an antigen, while T lymphocytes, which are categorized into one cluster of differentiation 4 (CD4) cells and one cluster of differentiation 8 (CD8) cells, identify small peptides as antigens, while inflammatory molecules, such as IL-7 and IL-5, play an essential part in T-cell homeostasis [26]. CD4, which acts as a co-receptor of the T-cell receptor (TCR), assists the latter through its communication with antigen-presenting cells, while both CD4 and the TCR complex bind to separate regions of the antigen-presenting MHC class II molecule, thereby helping to stabilize weak TCR interactions and enhancing TCR signaling [27].

Finally, it is important to underline that COVID-19 infection is characterized by a significantly better outcome in women, with male sex being considered a poor prognostic factor. A possible explanation for the latter may be the interaction between sex hormone levels and the immune system in women during reproductive age [28].

## 3. Hormone Treatment and the Immune System

Over the last two decades, numerous studies have been conducted to investigate the impact of hormone treatment on the immune system. The interactions between estrogen, progesterone, HRT, and the cells of the immune system as observed in women and in animal models are explored below.

### 3.1. Estrogen and the Human Immune System

A very interesting finding is that high estrogen levels in humans are associated with better vaginal immunization [29]. In vitro studies in humans similarly found that the presence of E2 promoted maturation of dendritic cells and differentiation of naïve CD4 T cells into T helper type 2 (TH2) cells [30]. On the other hand, it is reported that estradiol (E2) limits the efficiency of mature dendritic cells and their ability to stimulate T-cell proliferation [31].

Estrogen administration promotes production of Bcl-2, an anti-apoptotic molecule in B cells, and it is suggested that it subsequently increases the autoreactive resistance of B cells to apoptosis [32].

A recent study, moreover, demonstrated that estrogens lead to greater antibody affinity maturation by upregulating activation-induced deaminase (AID) and increasing the somatic hypermutation frequency and class-switch recombination [33].

### 3.2. Estrogen Treatment in Animals: Effects on Immune System

A large number of experimental data show that estrogens promote the protective Th1 response that follows parasitic infections. More specifically, in an animal model of parasitic infections, hormone treatment with 17-β estradiol (E2) in male mice resulted in higher expression of IFN-γ and lower levels of IL-10 in spleen cells [34]. The same effect was also noted in female ovariectomized mice treated with E2, which induced production of significantly higher levels of IFN-γ and antibodies [35], while HRT treatment in this model caused a lower degree of weight loss and of hematocrit diminution.

The protective role of estrogens has additionally been demonstrated in another animal study in which E2 treatment increased resistance to Toxoplasma gondii infections in female and also in male mice models [36].

In a mouse model, E2 administration to ovariectomized females resulted in a protective effect against HSV-2 infection [37]. The same protection was also observed in female rhesus macaques against transmission of SIV (simian immunodeficiency virus): this is a retrovirus that causes persistent infections in 45 or more species of African nonhuman primates [38]. In another murine HSV-2 challenge model, a study was made of the immune response after vaccination of ovariectomized females treated with E2. Ovariectomized females exhibited the same immune response to vaccination as unvaccinated controls, while the administration of E2 enhanced protection and significantly decreased disease severity [39]. However, the antibody titers in the E2-treated group were not found to be significantly higher compared to those of the control group, with only improved neutralization being reported.

It has, in addition, been determined that the estrogen-related receptor (ERR) effect on macrophages is necessary for the adequate production of interferon gamma (IFN-γ) and for an efficient immune response after infection with Listeria monocytogenes [40]. It is also observed that estrogens enhance B-cell activation and IgG production [41].

Concerning the effect of estrogens on T cells by use of peripheral blood mononuclear cells (PBMC), until now the reported data remain contradictory [42]. However, it is of note that in vitro studies point to a potential bias towards a Th2 response [43] and regulatory T (Treg) polarization [44] following E2 administration.

### 3.3. Progestogen and the Immune System in Humans and Animals

An association between Depo-Provera and increased incidence of chlamydia, HSV-2, HI, and HPV was confirmed with clinical studies in adult women [45]. On the other hand, progesterone is found to promote T-cell response [40], while it simultaneously diminishes the efficiency of dendritic cells (DCs) in the uptake of antigenic peptides [46]. Macrophages and DCs that were exposed to progesterone revealed a lower state of activation compared to that of untreated cells [47] and reduced apoptosis in NK cells [48]. Of note, progesterone is able to induce skewing of CD4 T-cell responses to Th2-type responses [49].

The same phenomenon was found in female animal models with prolonged exposure to progesterone, which resulted in increased susceptibility to HSV-2 genital infection [50], chlamydia trachomatis [51], SIV [52], and SHIV [53]. Moreover, progesterone treatment in mouse models reduced production of nitric oxide (NO) and macrophage activation [54].

### 3.4. HRT and the Immune System

In human studies, it was found that women taking HRT have higher lymphocyte numbers compared with post-menopausal women not receiving HRT [55]. This difference is already obvious within the first month after treatment initiation. There are also indications that HRT restores the levels of circulating monocytes in menopausal women to levels seen in women with menstruation [56].

HRT specifically counteracts the effects of menopause on B cells. It is hypothesized that the menopause brings about a large reduction in B cells, and specifically in B2 cell production; after administration of HRT B cells, production is increased [57]. By contrast, other human studies have demonstrated no effect of HRT on the menopausal changes to the T-cell line [55,58,59]. More explicitly, no improvement in the women’s health was observed with the reduction in the percentage of naïve T cells, the elevation of activated T cells, or the accumulation of memory T cells. However, this could possibly be attributed to the subjects’ biological aging rather than to menopausal transition. Also to be considered are the different types of hormonal therapy used in the various studies, which may, while relieving menopausal symptoms, also, to varying degrees, slow down the menopausal changes of the immune system.

The CD4/CD8 ratio of T cells decreases with age and is associated with reduced resistance to infection, while the percentage of NK cells is increased compared to that in young women. It is well-known that after menopause, this ratio is significantly reduced, together with the actual number of circulating B cells. HRT has been found to substantially neutralize these changes, while there are studies reporting that combined hormone therapy resulted in immediate increase of circulating B cells and especially in B2 population of B cells, as well as of T cells to a lesser extent [60,61]. Of special note, while estrogen therapy in hysterectomized women did not bring about a significant change in CD4 T cells or NK cells [62], in contrast, another study in menopausal nonhysterectomized women [63] following estrogen therapy demonstrated a reduction in CD8 T cells and a subsequent rise in the CD4/CD8 ratio, this revealing improved status of the immune system.

## 4. HRT and Specific Inflammatory Molecules

### 4.1. Human Studies

Menopause is in general characterized as an inflammatory state, which is mainly due to the increased levels of proinflammatory and inflammatory cytokines [2]. Various studies investigating the effect of HRT on inflammatory molecules have, however, differed significantly with regard to the regimen used for HRT (transdermal estrogen, tibolone), the daily dosage, and the duration of administration (3 to 6 months). Most of the data come from studies including mainly women in early postmenopause, a time period during which there is a continuous increase of FSH and decrease of estradiol—this lasting for about 2 years following the last menstrual period (STRAW+10 criteria) [64].

It has been established that in postmenopausal women, IL-1β, TNF-α, and IL-6 are significantly higher compared to levels in women of reproductive age [65]. HRT also increases the expression of IL-6 and soluble Il-6 receptor [66]; however, the latter menopausal inflammatory state characterized by increased cytokine concentrations is not detected or is noted to a lesser degree in women on estrogen therapy [67]. While the existing studies on inflammatory cytokines remain controversial, they nonetheless generally point to a decline in menopausal inflammation after HRT ( Table 1; Table 2).

More specifically, IL-6 levels are seen to have decreased in postmenopausal women receiving transdermal estrogen as soon as 3 months after the first prescription [68]. Therefore, it is suggested that IL-6 levels have a negative correlation with transdermal estrogens in HRT users, which is the same as that seen in women in menopause aged 40 to 65 years without any treatment. In another, smaller, human study though, IL-6 levels were found to be higher in women taking combined HRT compared to nonusers after PBMC stimulation by lipopolysaccharide (LPS) [67].

There are data showing no reversal of TNF-α increase in women who are on HRT [69], while levels of IL-6 are negatively correlated with serum estradiol concentration [70], leading to reduced inflammatory response with increased estradiol.

**Table 1:** Inflammatory molecule actions in menopausal women and after HRT use for menopause (NS: not studied).

Mediator	Molecular Weight (Da)	Menopause (Humans)	HRT (Hormone Replacement Treatment)	HRT Regimen	Dose	Duration	Study Participants
IL-1β	30,748	Increased [1]	No effect [70]	Low transdermal estrogen	1 mg/day	3 months	66
IL-4	17,492	Controversial data [1,67]	No effect [67]	Tibolone	2.5 mg/day	6 months	49
IL-6	23,718	Increased [1,62,68]	Decreased [68]	Low-dose transdermal estrogen	1 mg/day	3 months	66
IL-8	11,098	Increased [1,68]	NS				
IL-10	20,517	Increased [1,67]	No effect [66,67]	Transdermal estrogen	50 microg/day	3 months	30
				Tibolone	2.5 mg/day	6 months	49
IL-12	24,874	Increased [1,67]	No effect [67]	Tibolone	2.5 mg/day	6 months	49
TNF-α	25,644	Increased [1,62,67]	NS				
			No effect [67,70]	Low-dose transdermal estrogen	1 mg/day	3 months	66
				Tibolone	2.5 mg/day	6 months	49

The changes of IL-10 and IL-12 levels in menopausal transition are also not as yet fully clarified, with some studies reporting an increase and others a decrease during menopause [4]. However, HRT and transdermal estrogen have shown no effect on IL-10 levels [68,69]. With regard to IL-8, there is a negative correlation with estrogen levels in both human [70] and animal studies [71].

Another study demonstrated elevated markers of vascular repair along with improved microvascular reactivity following short-term administration of low-dose transdermal estradiol therapy in overweight/obese menopausal women, though no change was noted in levels of inflammatory molecules, including IL-1β, IL-6, MCP-1, and TNF-α [72].

IFN-γ is observed to be decreased in menopausal women and in women who have undergone bilateral oophorectomy [4]. After the initial treatment, IFN-γ increases [62]— while combined hormone therapy has been found to reduce IFN-γ production [73], this possibly attributable to progesterone’s opposing effect.

Tibolone, a synthetic steroid with estrogenic, progestogenic, and androgenic properties, is used widely as a treatment option in menopausal women. To date, there are, however, limited data concerning its effect on the immune system. In a human study, it was reported that tibolone had no effect on reversing the menopausal impact on IL-4, IL-10, IL-12, and TNF-α plasma levels [69], probably due to a combination of the above properties.

### 4.2. Animal Studies

In Table 2, we present all the data from animal studies that used primates, as well as menopause study models with other animals. It must be borne in mind that most animal models were rats, with different life expectancy and with mostly gonadectomy-induced menopause.

It has been noted that chronic E2 administration in ovariectomized animal models considerably increases the inflammatory cytokines IL-1β, IL-6, and TNF-α, and also of NO synthase in thioglycate-elicited macrophages [74], confirming previous reports that showed anti-inflammatory effects from short-term exposure to estrogens and enhanced production of proinflammatory cytokines in chronic exposure [75,76]. Furthermore, a recent study in ovariectomized rats reported that serum levels of TNF-α and IL-6 were increased, while treatment with estrogen or raloxifen or tamoxifen normalized these levels [77].

A study in ovariectomized rats showed that the increased levels of IL-6 and TNF-α were reduced after treatment with 17-β estradiol [78]. The same study reported that treatment with tibolone additionally reduced TNF-α, while there was no impact on IL-6 levels [78]. The same effect was also demonstrated in atrial natriuretic peptide (ANP) concentration, which was increased after HRT with tibolone, implying that tibolone has better anti-inflammatory effects compared to those of 17β-estradiol and could prevent cardiovascular disease.

**Table 2:** Inflammatory molecule actions in animal studies in menopause and after HRT use in menopause (NS: not studied).

Mediator	Menopause (Humans)	HRT (Animals)	HRT Regimen
IL-1β	Increased [1]	Decreased [75]	Estrogen, raloxifen, tamoxifen
IL-4	Controversial data [1,67]	NS	
IL-6	Increased [1,62,68]	Decreased [78]	Estrogen, raloxifen, tamoxifen
		No effect [78]	Tibolone
IL-8	Increased [1,68]	NS	
IL-10	Increased [1,67]	NS	
IL-12	Increased [1,67]	NS	
TNF-α	Increased [1,62,67]	Decreased [77,78]	Estrogen, raloxifen, tamoxifen, tibolone

## 5. Nutritional Supplementation with Flavonoids and the Immune System

Apart from hormone specimens, alternative approaches are additionally currently being developed for the alleviation of menopausal symptoms. Among them, nutritional supplementation with estrogen-like substances has, over the last decade, attracted a considerable amount of research interest.

Flavonoids are a class of polyphenolic secondary metabolites that are found in plants and are regularly consumed in the human diet. Until now more than 5000 flavonoids from various plants have been characterized. Based on their chemical structure, they are divided into subgroups, with the subgroup of isoflavones being the most commonly found, and are particularly important since they act as phytoestrogens in humans [79]. Isoflavones are mainly contained in soybeans, green beans, mung beans, cowpeas, kudzu roots, red clover sprouts, soy milk, peanuts (genistein), and green tea. The richest source of isoflavones is found in soybeans, with soy-derived foods and ingredients containing various concentrations of isoflavones.

In a human study of menopausal women taking either soy milk with 71.6 mg isoflavones or a supplement of 70 mg isoflavones for 16 weeks, an increase in circulating B cells was detected [80], while in an in vitro model, isoflavones resulted in increased IL-10 production [81]. The same trend of enhancement of the activities of cytotoxic T cells and NK cells was also observed in a human model using daidzein and genistein [82] and in a murine model [83] investigating the isoflavone genistein. Another animal study of ovariectomized female rats reported that nutritional supplementation with soybean or soybean and green tea enhanced the proliferative potential of B and T cells as well as of NK cell killing [84]. The same study, moreover, noted improved chemotaxis and macrophage production of reactive oxygen species (ROS), which have been demonstrated as being associated with ovarian function and estrogen levels [85].

Moreover, soymilk with 71.6 mg isoflavones or a supplement of 70 mg isoflavones for 16 weeks have been found to result in higher B-cell populations in healthy postmenopausal women, while the compounds have, in addition, been shown to have a protective role against DNA damage in menopausal women [80].

## 6. Isoflavones and Inflammation in Menopausal Women

There are data revealing a reduction in the inflammatory molecules IL-6 and TNF-α in women’s plasma levels after they received nutritional supplementation with isoflavones [86,87,88] (Table 3).

Chi et al., using soy isoflavones at a dose of 90 mg/day for 6 months, noted a reduction in the levels of IL-6 and TNF-α [86]. Moreover, Nadadur et al. also observed a reduction in levels of TNF-α, but no effect on IL-6 levels using food supplementation with either 50 mg isoflavones or 15 g soy protein in the form of tofu for 8 weeks [87]. Another study with 80 mg isoflavones (60.8 mg of genistein, 16 mg of daidzein, and 3.2 mg of glicitein) for 6 months showed reductions in TNF-α levels [88].

Recent in vitro studies have described the anti-inflammatory effects of isoflavones on peripheral blood mononuclear cells stimulated with lipopolysaccharide, thereby reducing IL1-β, IL-2, IL-6, and TNF-α levels [89,90].

**Table 3:** Inflammatory molecule actions in menopausal women and in vitro experiments after isoflavones use (NS: not studied).

Mediator	Molecular Weight (Da)	Menopause (Humans)	Isoflavones (Humans)	Isoflavones (In Vitro)
IL-1β	30,748	Increased [1]	No effect [91]	Decreased [89,90]
IL-4	17,492	Controversial data [1,67]	NS	NS
IL-6	23,718	Increased [1,52,68]	Decreased [86]	Decreased [89]
			No effect [87,91,92]	
IL-8	11,098	Increased [1,68]	No effect [93]	NS
IL-10	20,517	Increased [1,67]	NS	NS
IL-12	24,874	Increased [1,67]	NS	NS
TNF-α	25,644	Increased [1,62,67]	Decreased [86,87,88]	Decreased [89,90]
			No effect [80,91]	

Nevertheless, it must also be stressed that studies have been published showing no impact of this alternative approach on the immune systems of menopausal women. Moreover, a number of studies demonstrate no change in IL-2, TNF-α, and IFN-γ levels [80,91]. Among the latter are the study of Ryan-Borchers et al., who used either soy milk with 71.6 mg isoflavones or a supplement of 70 mg isoflavones for 16 weeks [80], and that of Beavers et al., who conducted a trial with soy milk for 4 weeks [91]. Other reports demonstrated no change in IL-6 levels with either 50 mg isoflavones or 15 g soy protein in the form of tofu for 8 weeks [87]; nor did the study by Huang et al., using soy milk with 112 mg isoflavones for 8 weeks, show any positive results [92]. Similarly concerning IL-8 levels, no change was detected after a supplement intake of 100 mg isoflavones (33 mg of genistein, 93.5 mg of dadzein, and 3.2 mg of glycitein) for 10 weeks [93]. The above data point to the fact that there is as yet insufficient evidence to support nutritional supplementation with isoflavones for enhancement of the immune response among menopausal women.

## 7. Conclusions

Taken together, the above-reported data reveal that the impacts of HRT on the immune system and its senescence remain to date unclear in many respects. Nevertheless, what appears evident is that HRT reverses the menopausal CD4/CD8 ratio and moderates the overall inflammatory state. Another beneficial effect is the fact that the increased levels of interleukins 1β, 6, 8, and TNF-α are reversed after HRT. As concerns other molecules, no effect of HRT has been observed on IL-4, 10, and 12.

The discrepancies noted between the available studies are partially attributable both to the large variety of current hormone replacement regimens, as these are shown in Table 1, and to the lack of consensus regarding the optimum immune parameters to be applied. It is thus necessary for more studies, both experimental and clinical, to be conducted in order to elucidate the observed alterations in concentrations in other mediators of the molecular pathways connecting HRT and the immune system.

An exact understanding of the impact of HRT and isoflavones on the functioning of the immune system will be the cornerstone for establishing targeted therapies for menopausal women. These future therapies will have the advantage of overcoming the effects of menopause without the side effects of the existing treatment options. At the same time, flavonoid-rich products, such as flavonoid-rich green tea, isoflavone-rich soy, flax seed, flavonol quercetin, and isoflavones, could be the basis for the development of pharmaceutical products and/or functional foods in the future.

Another interesting aspect is that targeted therapies may also improve the immune response to infection, inflammation, and vaccination of menopausal women, leading to the subsequent reduction of the consequent morbidity and mortality in this age group.

To conclude, an important focus of future research should be assessment of disease severity and mortality in menopausal women who have received either hormone therapy or flavonoids, compared to women who have had no treatment, or men of the same age group.
